# Treatment of Cerebral Vasospasm With Continuous Intra-Arterial Nimodipine: A Case Report

**DOI:** 10.7759/cureus.30507

**Published:** 2022-10-20

**Authors:** Susana Barbosa, Núria Jorge, Maria Luís Silva, Isabel Maia, Celeste Dias, Eduarda Pereira, José Artur Paiva

**Affiliations:** 1 Intensive Care Unit, Centro Hospitalar Universitário São João, Porto, PRT; 2 Neuroradiology, Centro Hospitalar Universitário São João, Porto, PRT; 3 Medicine, Faculty of Medicine, University of Porto, Porto, PRT

**Keywords:** case report, intra-arterial infusion, vasospasm, subarachnoid haemorrhage, intracranial aneurysm

## Abstract

Aneurysmal subarachnoid hemorrhage (aSAH) is an important cause of death and disability, not just due to the initial event, but also because of the delayed complications. Cerebral vasospasm (CV) stands out as a serious complication, with high prevalence and association with permanent neurologic impairment. The treatment of CV includes non-invasive measures, like oral nimodipine and induced hypertension, but also invasive measures. Endovascular rescue treatment (ERT), with intra-arterial approaches, is linked with improvement of cerebral perfusion and thus associated with a better outcome. There are several, widely studied substances used in intra-arterial approaches, none showing clear superiority over the others. The main issues with these substances are the adverse systemic effects and the recurrence of CV, due to the short duration of action. Recent studies suggest that the use of continuous infusion of nimodipine, instead of bolus injection, may be related to better outcomes. The authors present a case of severe refractory vasospasm successfully treated with continuous intra-arterial nimodipine infusion. A 23-year-old female was admitted with aSAH, Fischer IV, and Hunt Hess 5. A brain CT scan showed an extensive and diffuse subarachnoid hemorrhage causing ill-defined hypodensity of the brainstem, bilateral hemispheric hypodensities, and alterations compatible with diffuse cerebral edema. The cerebral angiography revealed an aneurysm in the emergence of the left posterior communicating artery. Coil target detachment was performed with partial occlusion of the aneurysm. On the fifth day of hospitalization, transcranial Doppler (TCD) ultrasonography revealed hemodynamic signs suggestive of vasospasm. Cerebral angiography performed later showed vasospasm of the terminal segment of the left internal carotid artery (ICA) and the A1 and M1 segments. Intra-arterial verapamil was instilled, with angiographic control showing a slight increase in the caliber of these segments. On the 13th day of hospitalization, the patient maintained sonographic evidence of vasospasm in the left ICA and middle cerebral artery (MCA). Selective catheterization of the left ICA was performed with a microcatheter at the level of the petrous segment and continuous infusion of 1 mg/h intra-arterial nimodipine was started. A progressive improvement was documented after the beginning of the continuous infusion of intra-arterial nimodipine, which was maintained for five days, and angiographic control revealed improvement of vasospasm in the terminal portion of the ICA as well as in the A1 and M1 segments. Long-term continuous intra-arterial nimodipine infusion is a promising technique for the treatment of refractory CV and may be considered in selected cases.

## Introduction

Aneurysmal subarachnoid hemorrhage (aSAH) is an important health problem with an incidence of 9 per 100,000 and a mortality rate of about 60% within six months [1]. This devastating outcome is related to the initial injury, however, late complications can aggravate it [2]. Cerebral vasospasm (CV) is a serious consequence of aSAH, occurring in approximately 70% of all patients, and 7% will develop delayed ischemia [3], which may result in permanent neurological impairment [2].

The established treatments for a CV include oral nimodipine and induced hypertension [4-5]. In some preliminary studies, IV milrinone seems to be associated with a lower rate of endovascular angioplasty and a positive impact on long-term neurologic [6]. Endovascular rescue treatment (ERT) with intra-arterial approaches may improve cerebral perfusion and the outcome of patients with critical CV. Multiple substances have been studied for intra-arterial administration, including milrinone, verapamil, nicardipine, papaverine and colforsin, with variable results.

Mayer et al. are credited for the concept of continuous infusion of nimodipine to overcome the drawbacks of bolus injection [7]. Long-term continuous intra-arterial nimodipine infusion is a promising technique for the treatment of refractory CV [8-10].

The authors present a case of a 23-year-old female with an aSAH who developed severe vasospasm, refractory to usual treatment that was treated with continuous intra-arterial nimodipine infusion in a neurocritical care unit of a tertiary hospital intensive care department. This intensive care unit (ICU) admitted 65 patients with SAH between January 2018 and December 2019, 38.5% with a Hunt and Hess score of 4 or 5 and 89.2% with a Fisher score of 3 or 4, with an ICU mortality of 10.8%.

## Case presentation

A 23-year-old female, with a history of migraine since the age of 8, was admitted to the emergency room after a sudden deterioration of consciousness. In the pre-hospital evaluation, a generalized tonic-clonic seizure was described, with a left conjugated shift of the gaze, with a post-crisis Glasgow Coma Score of 3 and she was sedated and intubated.

**Figure 1:**
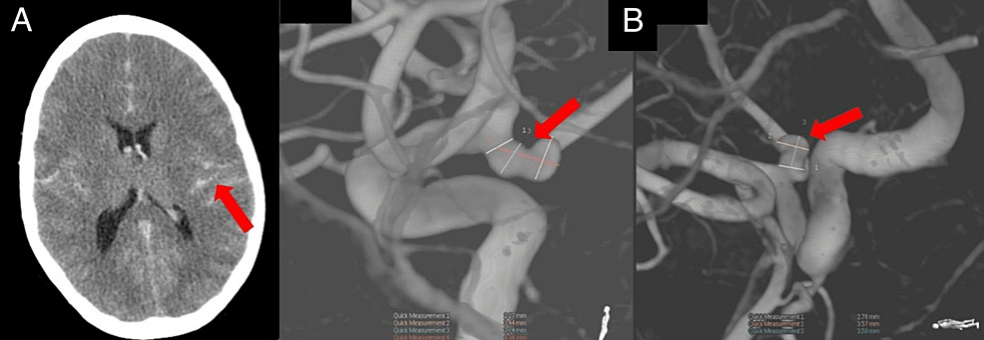
A: Cerebral CT scan on admission showed extensive SAH and diffuse hypodensities compatible with global cerebral edema. B: Cerebral angiography at admission identified an elongated bilobed aneurysm in the left posterior communication artery. SAH, subarachnoid hemorrhage

The initial CT scan of the brain showed extensive and diffuse subarachnoid hemorrhage causing ill-defined brainstem hypodensity, bilateral hemispheric hypodensities, and alterations compatible with diffuse cerebral edema (Fischer IV, Hunt Hess 5) (Figure 1A). Cerebral angio CT revealed an aneurysm in the emergence of the left posterior communicating artery.

Cerebral angiography identified an elongated bilobed aneurysm located in a hyperplastic branch above the left posterior communicating artery, wide neck (2.87 mm), and fundus of 3.45 mm (Figure 1B).

**Figure 2:**
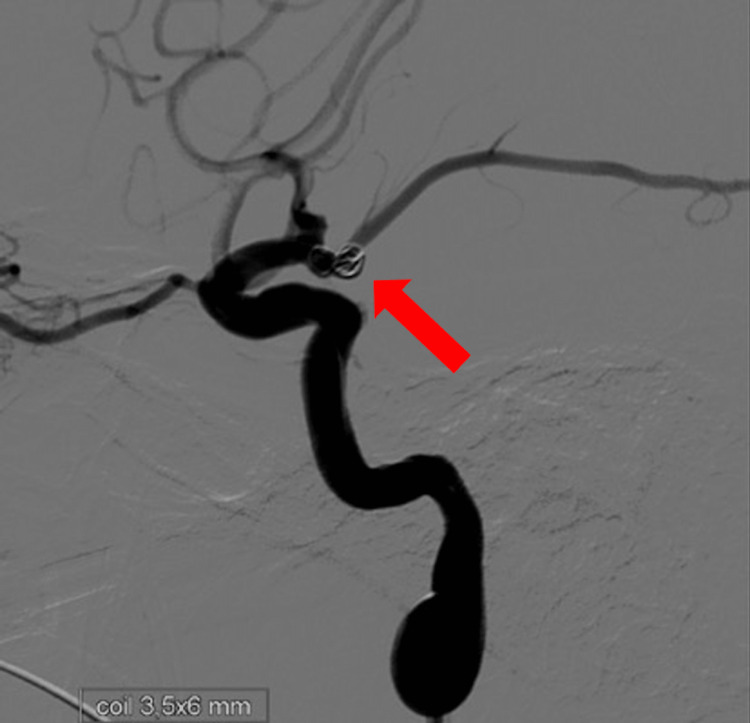
Cerebral angiography: coil target with partial occlusion of the aneurysm.

Coil target detachment was performed with partial occlusion of the aneurysm (Figure 2).

She was admitted to the neurocritical care unit after the procedure, deeply sedated and under invasive mechanical ventilation. Intracranial pressure (ICP) and cerebral oxygenation (PbtO2) sensors were inserted. Vasopressor support (noradrenaline) to maintain cerebral perfusion pressure (CPP) and oral nimodipine were started. 

On the third day of hospitalization, she developed progressive increase of ICP (maximum value 29 mmHg). Brain CT scan showed redistribution of intracranial subarachnoid hemorrhagic content and a slight increase in the amplitude of the ventricular system. Due to intracranial hypertension refractory to osmotic therapy (hypertonic saline solution), an external ventricular drain (EVD) was placed, with subsequent ICP control and brain tissue oxygen tension (PbtO2) >20 mmHg.

On the fifth day of hospitalization, transcranial Doppler (TCD) ultrasonography was suggestive of vasospasm of the anterior cerebral artery (ACA), terminal left internal carotid artery (ICA), and proximal segment of the middle cerebral artery (MCA). The cerebral angiography performed later did not reveal significant vasospasm in the territories of the right ACA and MCA, however, it showed filling of the left A2 segment via the anterior communicating artery and vasospasm of the terminal segment of the left ICA and the A1 and M1 segments. Intra-arterial verapamil was instilled, 10 and 30 mg respectively, with angiographic control showing a slight increase in the caliber of these segments. To optimize cerebral blood flow, systemic milrinone (1 mcg/kg/min) was added without a significant increase in the norepinephrine doses.

Serial TCDs were performed (Table 1), with sonographic changes suggestive of vasospasm, confirmed by cerebral angiography, with imaging control showing improvement after local verapamil.

**Table 1:** TCD during hospitalization. TCD, transcranial Doppler; Vs, systolic velocity; Vd, diastolic velocity; PI, pulsatility index; HI, hemispheric index

TCD				Day 5	Day 11	Day 12	Day 13	Day 14	Day 15	Day 18
											Carotid	Right	A1	Vs (cm/s)	186	191	176	199	150	157	131
Vd (cm/s)	103	109	100	119	88	81	82						
PI	0,6	0,6	0,6	0,5	0,6	0,7	0,5						
HI	3.5	2,7	3,3	3,2	3,5	2,4	2,6						
Left	M1	Vs (cm/s)	238	228	278	273	114	131	123		
		Vd (cm/s)	133	96	199	180	53	73	86		
		PI	0,6	0,9	0,4	0,4	0,8	0,6	0,4		
		HI	3,8	4	6,2	5	2,4	2,2	1,7		

**Figure 3:**
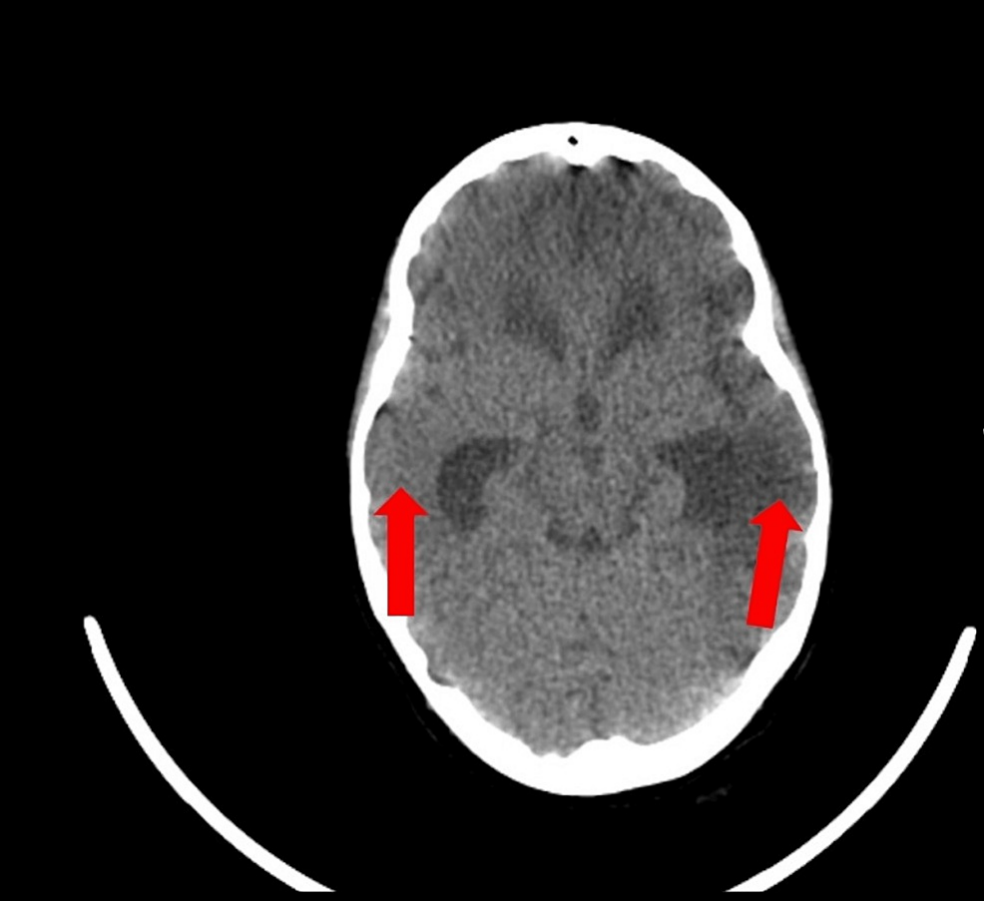
Cerebral CT scan revealed ischemic lesions in the right temporal and left hemispheric cortico-subcortical topography.

Brain CT revealed ischemic lesions in the right temporal and left hemispheric cortico-subcortical topography, with the deep territory of the left MCA and the frontal lobe partially preserved (Figure 3).

**Figure 4:**
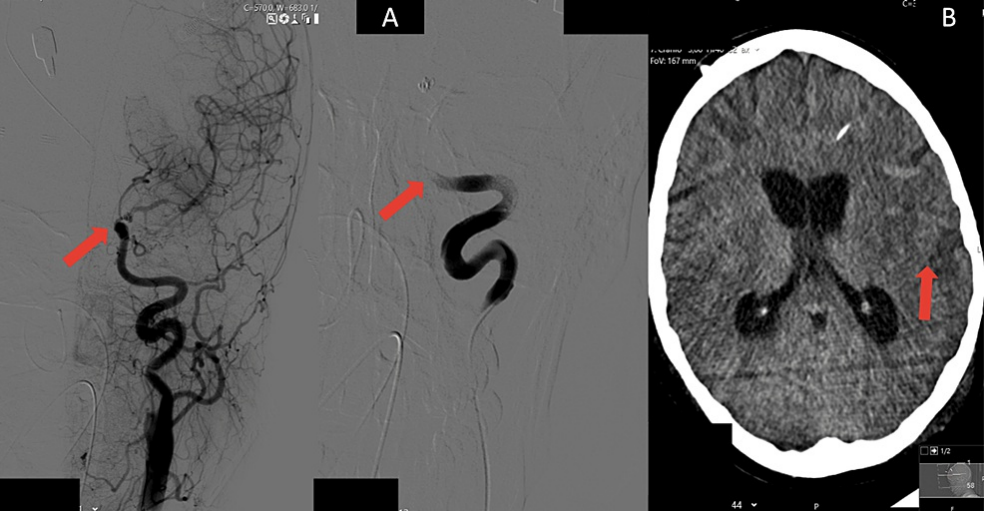
A: Selective catheterization of the ICA was performed with a microcatheter at the level of the petrous segment with evidence of marked vasospasm. B: Cerebral CT scan showed less conspicuity of diffuse cortico-subcortical edema areas, without evidence of new parenchymal ischemic areas. ICA, internal carotid artery

Between the 5th and 13th day of hospitalization, milrinone was maintained and daily TCD was performed, at which point, sonographic evidence of vasospasm in the left ICA and MCA (Table 1), with apparent inversion of the flow direction in the left A1 segment was detected and selective catheterization of the left ICA was performed with a microcatheter at the petrous segment level, with evidence of marked vasospasm (Figure 4A) of this axis and continuous infusion of 1 mg/h intra-arterial nimodipine through the microcatheter was started. Concomitantly, hypocoagulation with unfractionated heparin was started by targeting an activated partial thromboplastin time (aPTT) of 50-60 s.

Continuous hemodynamic monitoring was maintained and CPP was optimized according to the pressure reactivity index (PRx). Since the beginning of the continuous infusion of intra-arterial nimodipine through the microcatheter, which was maintained for five days, she showed progressive improvement, with TCD on the 14th day of hospitalization (first after continuous infusion of intra-arterial nimodipine) showing an overall decrease in velocities, with the A1 segment of the left ACA to present flow in the physiological direction (Table 1).

On the 18th day, catheterization of the left ICA was performed with images suggesting long arterial dissection, but without relevant repercussion on the luminal caliber, improvement of vasospasm in the terminal portion of the ICA as well as in the A1 and M1 segments, but evidence of severe focal stenosis (>90%) in the proximal portions of M2. Administration of verapamil (28 mg) in the left ICA led to angiographic improvement in all left carotid axis segments. The microcatheter was removed and unfractionated heparin was suspended. 

Brain CT showed less conspicuity of diffuse cortico-subcortical edema areas, without evidence of new parenchymal ischemic areas (Figure 4B).

**Figure 5:**
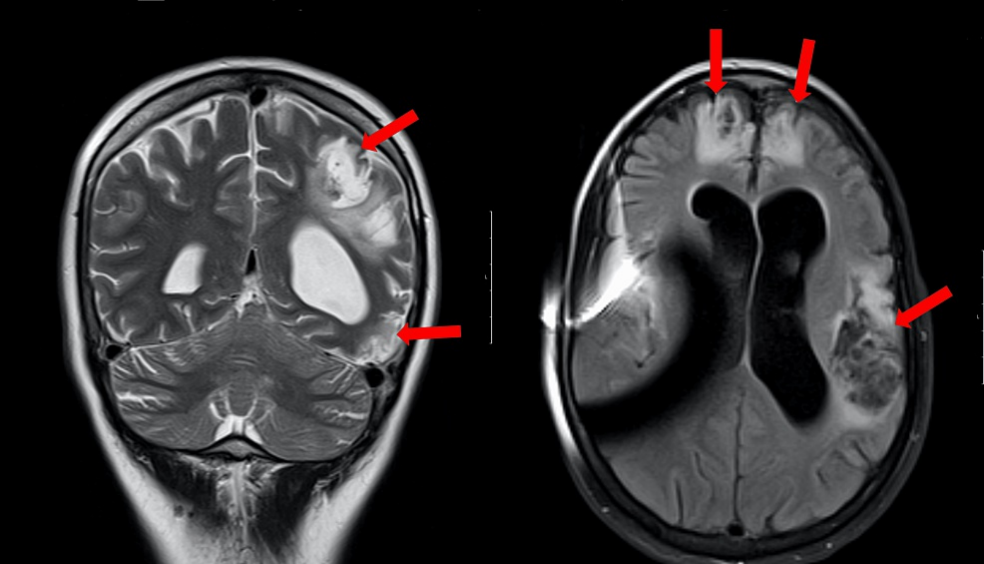
Cerebral MRI showed ischemic lesions in different evolutionary stages in both ACA and MCA territories. ACA, anterior cerebral artery; MCA, middle cerebral artery

Nimodipine was suspended on the 21st day after aSAH and the patient was weaned from sedation. On the 25th day after aSAH, cerebral MRI showed the presence of ischemic lesions in different evolutionary stages, namely, in the left striated nucleus, radiated corona and cortico-subcortical planes of paramedian frontal region; frontal-temporo-parietal cortico-subcortical ischemic lesions on the left MCA territory; anterior parasagittal frontal cortico-subcortical ischemic lesions, in the territory of both ACAs; and in the territory of the right MCA, including the insula (Figure 5). ICP and PbtO2 sensors were removed and, on the 27th day of hospitalization, EVD was removed.

**Figure 6:**
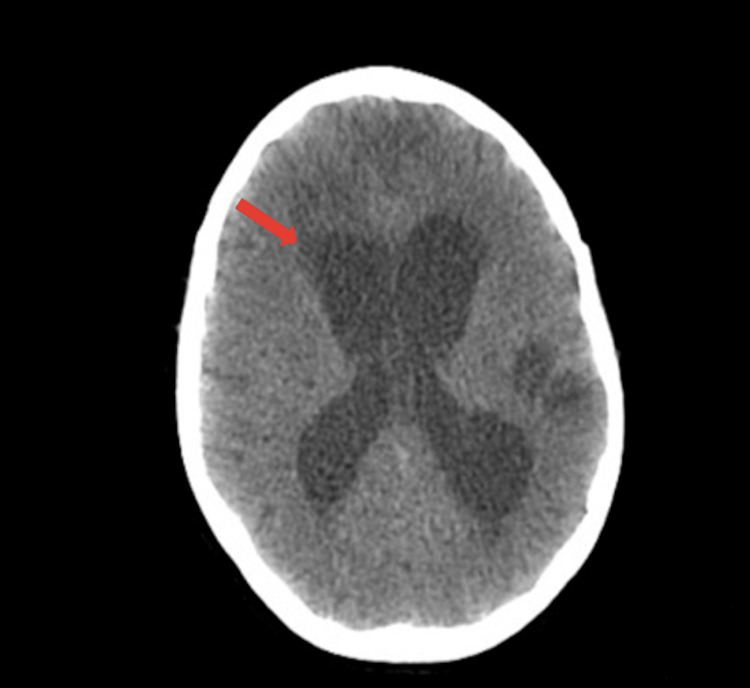
Cerebral CT scan showed persistent hydrocephalus.

After suspension of sedation, the patient presented, predominantly motor aphasia, spontaneous eye-opening, with gaze direction, carrying out orders inconsistently and right hemiparesis with brachial predominance. She was extubated on the 31st day of hospitalization and, on the 33rd day after admission, she was transferred to the Neurosurgery ward. Control brain CT scan showed persistent hydrocephalus and surgery for ventriculo-peritoneal shunt insertion was performed (Figure 6).

On the 55th day, she was discharged to a rehabilitation center. At hospital discharge, she was calm, cooperative, and able to carry out orders inconsistently but with psychomotor lentification. She presented dysphasia, predominantly motor aphasia and right hemiparesis with brachial predominance (mRankin 4). She remained for three months in the rehabilitation center, after which she maintained rehabilitation at home.

Reassessment in follow-up consultation of our Intensive Care Department revealed progressive motor and cognition neurological improvement. Eighteen months after hospital discharge, she can now walk autonomously, self-care, although with less strength in her right upper body limb, and she is able to do complex tasks and interact socially with family and friends (mRankin 2).

## Discussion

The currently accepted treatment options for CV are: prophylactic oral nimodipine [11] and vasopressor-induced hypertension. The second, however, has been recently challenged [12].

Regardless of these therapies, some patients develop persistent hypoperfusion. To avoid potentially catastrophic consequences, ERT is considered case-to-case as a rescue treatment, despite having no high-level evidence. Two techniques have been established: 1) transcutaneous balloon angioplasty (TBA), used in proximal vessel narrowing detected on digital subtraction angiography and 2) intra-arterial application of vasodilating substances, used in distal vessel constriction, inaccessible to TBA. Both treatments have pitfalls. TBA has the risk of dissection or rupture that can result in permanent vessel wall alteration with dysfunction of autoregulation of unclear consequences [13-14]. Intra-arterial administration of vasodilators, although predominantly concentrated locally [15], has potential hypotensive effects as well as the risk of vessel injury, thrombosis, or hemorrhage.

Furthermore, the vasodilating substances that are commonly used have a short half-life, allowing the recurrence of severe vasospasm in sedated patients in which clinical monitoring is impossible.

Various substances have been used in clinical practice to control CV. Milrinone and papaverine, with similar action mechanisms, have little or no significant systemic effects on arterial blood pressure, but the recurrence rate of CV after intra-arterial bolus injection is more than 20% [16]. The intra-arterial infusion of verapamil dilates spastic cerebral arteries [17], but high doses and several hours of infusion are required [18-19]. The advantage of verapamil over nimodipine is not evident. However, the reported data shows a 30% angiographic failure rate, probably by the short action duration of such bolus [10, 20].

In the series published by Musahl et al. [21], continuous local intra-arterial nimodipine administration (CLINA) was considered for patients with severe symptomatic vasospasm despite IV nimodipine infusion and triple-H therapy when the pattern of CV prohibited TBA. Advantages of CLINA were: 1) the prolonged effect on CV, lasting several days; 2) the possibility of reaching not only proximal spastic vessel segments but also distal portions of the affected vascular territory; and 3) the possibility of treating diffuse distal vasospasm. Potential complications included thromboembolic infarction, vessel dissection, air emboli, kinking or thrombosis of the microcatheter, induced arterial hypotension, and sepsis [21].

Specific indications of this technic are peripheral, diffuse, and multilocular vasospasm that can be unilateral or bilateral, although there are not enough studies to prove its benefits on the outcome of this high-risk group of patients [21].

## Conclusions

Our case reports an aSAH in a 23-year-old female who developed severe vasospasm, refractory to the usual treatment that was treated with CLINA, starting on the 13th day, and with the duration of five days, allowing the interruption of the cerebral ischemia. This case report shows that CLINA is a safe and efficient treatment of severe CV when conservative treatments fail. However, further studies are needed. There are no guidelines for patient selection, dosage of nimodipine or when to initiate CLINA and its duration. Providing a treatment algorithm, prompt ERT, in selected patients may be a safe and effective treatment in those patients were conservative measures failed.
